# Multipod Bi(Cu_2-x_S)_*n*_ Nanocrystals formed by Dynamic Cation–Ligand Complexation and Their Use as Anodes for Potassium-Ion Batteries

**DOI:** 10.1021/acs.nanolett.2c03933

**Published:** 2022-12-06

**Authors:** Nilotpal Kapuria, Sumair Imtiaz, Abinaya Sankaran, Hugh Geaney, Tadhg Kennedy, Shalini Singh, Kevin M. Ryan

**Affiliations:** Department of Chemical Sciences and Bernal Institute, University of Limerick, V94T9PXLimerick, Ireland

**Keywords:** Heterostructures, Metal/semiconductor, Potassium ion battery, Ligands, Intermediates, Catalyst-assisted, Seeded-growth

## Abstract

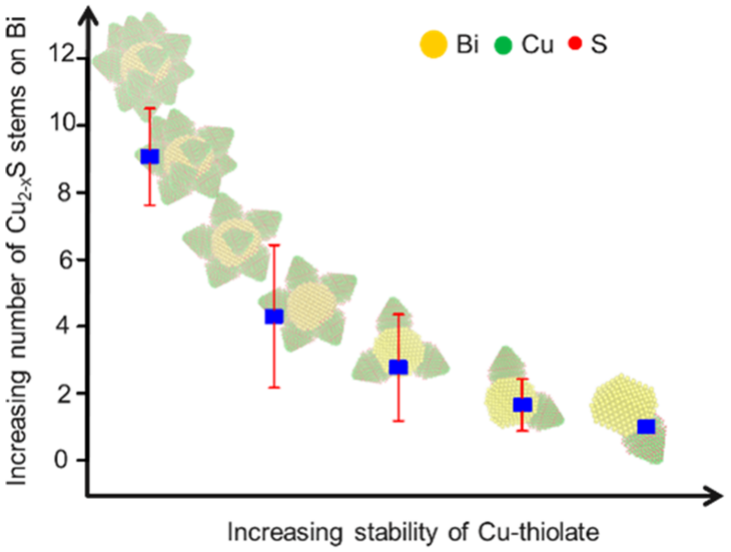

We report the formation of an intermediate lamellar Cu–thiolate complex, and tuning its relative stability using alkylphosphonic acids are crucial to enabling controlled heteronucleation to form Bi(Cu_2-*x*_S)_*n*_ heterostructures with a tunable number of Cu_2-*x*_S stems on a Bi core. The denticity of the phosphonic acid group, concentration, and chain length of alkylphosphonic acids are critical factors determining the stability of the Cu–thiolate complex. Increasing the stability of the Cu–thiolate results in single Cu_2-*x*_S stem formation, and decreased stability of the Cu–thiolate complex increases the degree of heteronucleation to form multiple Cu_2-*x*_S stems on the Bi core. Spatially separated multiple Cu_2-*x*_S stems transform into a support network to hold a fragmented Bi core when used as an anode in a K-ion battery, leading to a more stable cycling performance showing a specific capacity of ∼170 mAh·g^–1^ after 200 cycles compared to ∼111 mAh·g^–1^ for Bi–Cu_2-*x*_S single-stem heterostructures.

Heterostructure nanocrystals (NCs) with two or more chemically and structurally distinct domains can exhibit enhanced physical and electronic properties due to the synergistic effects of the ensemble.^1−5^ The synthetic development of these NCs has enabled significant advances in catalytic, optoelectronic, thermoelectric, battery, and biomedical applications.^6−14^ A general approach to synthesizing colloidal heterostructure nanocrystals is to employ presynthesized metal or semiconductor NCs as seeds.^15−19^ Typically, secondary phases nucleate on the seed, with the change of Gibbs free energy of the surface and epitaxial relation of the crystal domains deciding the architectural outcome of secondary growth. Semiconductor seeds exhibiting high ionic conductance (e.g., Cu_2-*x*_S) allow heterointerface formation with an immiscible phase via topotactic cation-exchange.^20,21^ However, direct growth methods for multicomponent heterostructures are underdeveloped due to the difficulty in controlling the delicate reactivity balance of multiple precursors. In solution–liquid–solid (SLS) growth, liquid-metal droplets are used to catalyze the growth of semiconductor NCs, and the NC growth kinetics can be regulated by changing the nature (solid or liquid) of the metal seeds (Bi, In, and Sn). However, the morphology of SLS grown NCs is limited to 1D, and SLS has not been explored in the direct growth of complex heterostructures with 3D morphology. Recent findings on inorganic NC growth indicate that prenucleation intermediates formed between precursors and ligands play a crucial role in determining the morphology and size dispersity of unary and binary NCs.^22−24^ These intermediaries typically present as molecular clusters, magic-sized nuclei, mesophases, or lamellar structures as the immediate source of monomers.^24−27^

Harnessing the beneficial properties of different dimensionalities within hybrid morphologies allows the limitations of individual domains to be bypassed. In electronic applications, a 0D aspect delivers a short charge transport length, while a 1D structure component can allow fast charge transport along the long-axis; when combined, the heterostructure can display efficient charge separation. Notably, heterostructures with branched morphologies possessing additional free space between branches are of interest to alkali metal ion batteries, mainly due to the advantages of stable solid electrolyte interface (SEI) layer formation, short ionic diffusion channels, and a buffer network to tackle volume expansion.^28,29^ K-ion batteries (PIB) are a promising alternative to LIBs due to the higher natural abundance of K-resources (1.5 wt %), low-cost, and a comparable standard reduction potential of K^+^/K to Li^+^/Li.^30^ However, due to the much large ionic radius of K^+^ (0.138 nm), alloying type anodes based on Bi and Sb NCs experience a vast volume expansion during alloying, resulting in the pulverization of the active materials.^31,32^ An alternative set of anode candidate materials that operate via conversion-based mechanisms have been studied in parallel to alloying materials, with branched structures and porosities allowing for enhanced performance.^33,34^ Considering the similar material design strategies for alloying and conversion materials to mitigate volume expansion related issues, nanostructure design with an alloying type core can provide high-energy density. However, the peripheral conversion type arms can evolve into a support matrix embedding the alloying material to provide structural integrity.

Here, we demonstrate the direct SLS growth of Bi(Cu_2-*x*_S)_*n*_ heterostructures with tunable Cu_2-*x*_S stem formation. We show that a Cu–thiolate complex forms as a reaction intermediate and controls Cu^+^ supply during heteronucleation. The stability of this Cu–thiolate complex was modulated *via* the systematic variation of the alkylphosphonic acid concentration, alkyl chain-length, and denticity of phosphonic acid group. Crucially, the alkyl chain-length and number of phosphonates groups are critical to controlling the Cu–thiolate stability. Stability modulation affects the Cu^+^ supply during heteronucleation, thereby allowing the number of Cu_2-*x*_S stems formed on the Bi core to be tuned. Furthermore, the electrochemical performance of the Bi–Cu_2-*x*_S based anodes for KIBs was examined. The morphological advantage of multiple stem formation is demonstrated by comparing the electrochemical performance of single and multistem anodes. Amorphization of multiple Cu_2-*x*_S stems forms a network to support the fragmented Bi core, resulting in enhanced cycling stability and rate capability with higher specific capacity (∼170 mAh·g^–1^ after 200 cycles) compared to single pods (∼111 mAh·g^–1^ after 200 cycles).

Bi–Cu_2-*x*_S heterostructures were prepared using a colloidal hot-injection approach with Cu(acac)_2_, and BiCl_3_ as metal precursors in oleylamine (OLA) and octadecene as solvents and 1,2-ethylenediphosphonic acid (EDPA) (0.1 to 0.5 mmol) as a ligand. A mixture of *tert*-dodecylmercaptan and 1-dodecanethiol was used as the sulfur source and injected into the solution of cationic precursors and solvent mixture at 135 °C with subsequent heating to 160 °C (detailed procedure described in the Supporting Information). Characterization of aliquots withdrawn at various stages of the reaction helped to decipher the nucleation and growth mechanism of these heterostructures. Upon adding the S-source, the blue color of the reaction solution turns orange, indicating the formation of the lamellar Cu–thiolate complex (Figure S1). The presence of lamellar Cu–thiolate can be observed in the aliquot withdrawn at 140 °C (Figure 1a). Subsequently, the color of the reaction solution changes to gray, finally leading to black, indicating the heteronucleation of Cu_2-*x*_S on in situ generated Bi seeds. The average size of initially formed Bi NCs is below ∼3 nm causing a melting point depression of Bi to below 150 °C (Figure 1b, Figure S2a) which catalyzes the SLS growth of Cu_2-*x*_S.^19,35^ Heterostructure formation begins with a single Cu_2-*x*_S stem (hereon referred to as “pod”) as shown in the nascent heterostructures in Figure 1c and Figure S2b. The Cu_2-*x*_S pod is present in the monoclinic phase, which is corroborated by the *d*-spacing values of 3.2 Å for  and  planes interfacing with the rhombohedral Bi seed (Figure 1d). ^1^H NMR analysis of the 140 °C aliquot provides information about the reactions occurring before heteronucleation (Figures S3 and S4). A peak at ∼3.2 ppm results from the ketimine (**1**) formed due to the reaction of acetylacetone generated from Cu(II)–acetylacetonate and primary aldamine formed upon *β*-hydrid elimination of OLA (Figure 1e, plausible reactions and full spectra of ^1^H NMR are in Figure S3). Weak peaks present at ∼7.6 and ∼3.3 ppm from secondary aldamine (**2**, **3**) and the peak present at ∼10.9 ppm from −OH proton of enolimine (**5**) confirms the reducing nature of OLA (Figure 1c).^36^ The *α*-proton peak (∼2.6 ppm, 4) from the disulfide formed upon alkanethiol oxidation is observed beside the *α*-proton peak from the metal-coordinated OLA (shifted downfield to ∼2.8 ppm). Thus, the OLA oxidizes to primary aldamin to provide two electrons, and alkanethiol oxidizes to disulfides by giving up two electrons.^37,38^ Hence, *in situ* formation of Bi NCs initiates with transient Bi–oleylamido complex formation from OLA and BiCl_3_ in the solution, and introducing the reducing agent dodecanethiol (DDT) ensures their subsequent reduction to Bi^0^.

**Figure 1:**
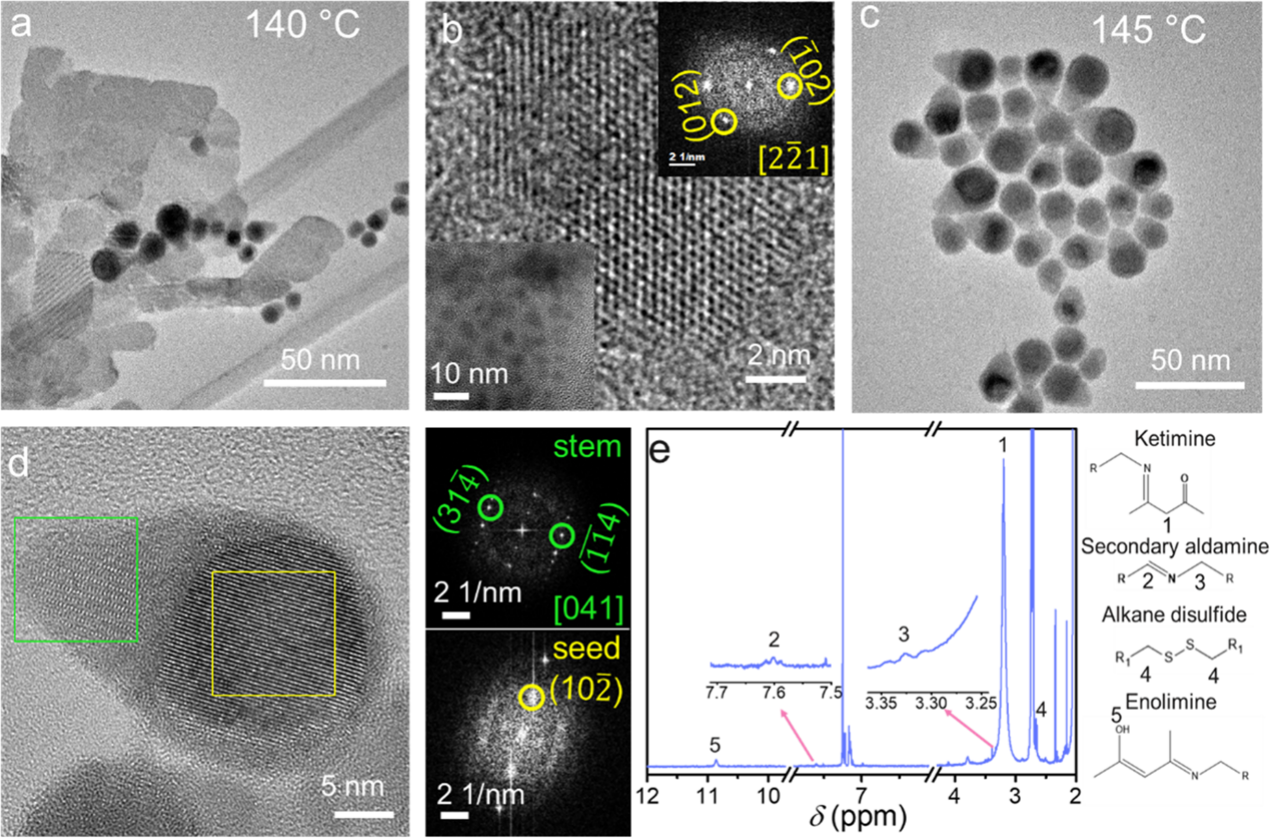
Transmission electron microscopy (TEM) image of aliquot samples withdrawn at 140 °C showing (a) lamellar Cu–thiolate with the nascent Bi–Cu_2-*x*_S NCs, and (b) high resolution TEM (HRTEM) of Bi NCs with selected area fast Fourier transform (FFT) pattern and low magnification TEM (LMTEM) in insets (c) LMTEM of Bi–Cu_2-*x*_S heterostructures collected at 145 °C. (d) HRTEM of a single Bi–Cu_2-*x*_S heterostructure with FFT patterns of Bi seed and Cu_2-*x*_S stem. (e) ^1^H NMR of aliquot withdrawn at 140 °C in CDCl_3_. The reference peak of chloroform is at 7.26 ppm.

The aliquots withdrawn below 150 °C displayed only single pods where the pod length changes based on the temperature. If the heterostructures are held for 5 min to grow at 150 °C, multipod formation starts (Figure S5). With the increment in time and temperature, the number of pods increased where a low concentration of EDPA (0.1 mmol) is used, forming Bi–Cu_2-*x*_S heterostructure multipods (Figure 2a). For a higher concentration of EDPA (≥0.25 mmol), the number of pods significantly reduces as for 0.25 mmol, the number of pods ranges between 1 and 3 (Figures 2b and S11a), and a higher concentration of 0.5 mmol of EDPA ensures only a single pod formation (Figure 2c). In addition to reducing the number of pod formations, the Bi seed becomes faceted. Additionally, changing the phosphonic acid to 1,6-hexylenediphosphonic acid (0.1 mmol) results in mixed population of multipods and single pods (Figures S6 and S11b). Furthermore, usage of a short-chain (number of carbons <8) alkylmonophosphonic acid such as hexylphosphonic acid (0.1 mmol) also results in mixed population pods formed on the Bi core (Figures S6 and S11b). In contrast, long chain (number of carbons >8) alkylphosphonic acids (0.1 mmol) facilitates single pod formation. Hence, the number of phosphonic acid groups, the chain-length and concentration of alkylphosphonic acids are crucial to alter the number pods formed.

**Figure 2:**
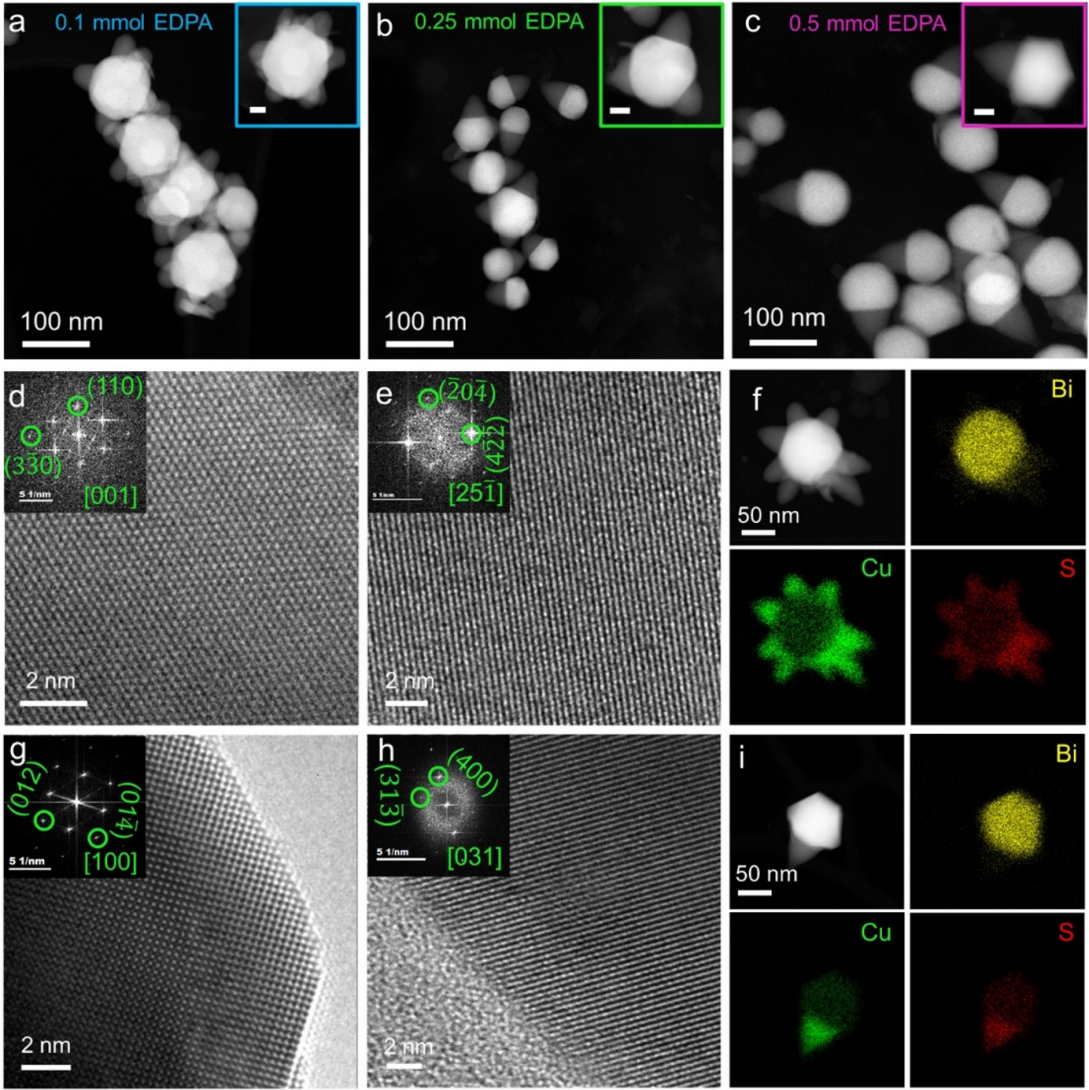
Scanning transmission electron microscopy-annular dark field (STEM-ADF) micrographs of Bi–Cu_2-*x*_S heterostructures with pod numbers ranging from multi to single for an EDPA concentration of (a) 0.1 mmol, (b) 0.25 mmol, and (c) 0.5 mmol with the inset showing individual heterostructures with a scalebar of 20 nm. HRTEM of (d) Bi seed and (e) Cu_2-*x*_S stem in Bi–Cu_2-*x*_S heterostructure multipods. (f) STEM-ADF micrographs of Bi–Cu_2-*x*_S heterostructure multipods accompanied by STEM–energy dispersive X-ray spectroscopy (STEM-EDS) element maps for Bi (yellow), Cu (green), and S (red). HRTEM of (g) faceted Bi seed and (h) Cu_2-*x*_S stem in Bi–Cu_2-*x*_S heterostructure single pod. (i) STEM-ADF micrographs of Bi–Cu_2-*x*_S heterostructure single pod accompanied by STEM-EDS element maps for Bi (yellow), Cu (green), and S (red).

In the multipod, the Bi seed is present in the rhombohedral (*R*3̅*m*) phase, *d*-spacing values of 2.3 and 1.3 Å for (110) and  planes of the seed match with metallic Bi (Figure 2d). The stem of the multipod is monoclinic Cu_2-*x*_S with *d*-spacing of 2.4, and 3.3 Å for () and () facets respectively calculated from the HRTEM and the corresponding FFT (Figure 2e). The STEM-EDS elemental mapping of the heterostructure multipods displays the presence of a Cu- and S-rich stem anproblemsd a Bi-rich head (Figure 2f). Similarly, in the single pod, the Bi-seed is present in the rhombohedral (*R*3̅*m*) phase and exhibits *d*-spacings of 3.3 and 2.4 Å for (012) and  facets (Figure 2g). The *d*-spacing of 3.4 Å corresponding to the (400) plane of the stem of single pod confirms the monoclinic Cu_2-*x*_S phase (Figure 2h). STEM-EDS elemental mapping of the heterostructure single pod further confirms the presence of Cu and S in the stem and Bi in the seed (Figure 2i).

The orange product (Figure S12) obtained after precipitation of 140 °C aliquots display periodically spaced (00*n*) reflections in the XRD pattern from lamellar Cu–thiolate (Figure 3a). The bilayer spacing in the lamellar-framework changes from ∼37.1 Å for 0.1 mmol of EDPA to ∼35.6 Å for 0.5 mmol of EDPA. Hence, a decrease of ∼1.5 Å in bilayer spacing is observed with increased EDPA concentration. The distance of ∼37.1 Å is in accordance with a bilayer-framework involving DDT, which possesses a chain-length of 17.7 Å when fully extended.^39^ The decreased spacing for increased concentration of EDPA can be attributed to replacing some of the long-chain DDT with EDPA possessing a shorter carbon chain-length resulting in the relatively reduced average distance between layers. The shift of peaks in the low angle XRD pattern of Cu–thiolate toward higher angles with increased EDPA concentration is consistent with the replacement of long-chain DDT with short-chain EDPA. The DSC thermogram (Figure 3b) of the Cu–thiolate complex showed an endothermic peak around 143 °C for 0.1 mmol of EDPA, which is shifted to 147 °C for 0.5 mmol of EDPA. The presence of the endothermic peak can be ascribed to the melting of crystalline lamellar structure into mesophase structure facilitating higher concentration of Cu^+^ to nucleate.^26^ The presence of the *β*-proton peak from Cu^+^-coordinated ketimine at ∼4.2 ppm in the ^1^H NMR spectra of 145 °C aliquot further suggests the melting of Cu–thiolate occurs <145 °C when 0.1 mmol EDPA is used (Figure S4). When higher concentration of EDPA is used, the increased melting temperature displays the increased stability of the lamellar Cu–thiolate phase indicating that a low amount of Cu^+^ is available for heteronucleation at a temperature ∼140 °C. In the Cu–thiolate complex, the x-type phosphonate group will coordinate with Cu^+^. The ∼6 ppm upfield shift in the ^31^P NMR of the aliquots compared to neutral EDPA confirms the direct interaction of phosphonate ligands with Cu^+^ of the Cu–thiolate (Figure 3c).^40,41^ Considering the high P*k*_a3_ value (>7.5) of diphosphonates and presence of excess OLA, at least two protons will be always available to form hydrogen bonds.^42^ We hypothesize that the hydrogen-bond forming tendency between DPAs could stabilize the lamellar structure chains when present in higher concentration (Figure 3d).^43^ However, the low concentration of DPA is not enough to form a stable hydrogen-bonding network to enhance the stability of the lamellar-complex. Additionally, x-type phosphonate groups interfere with the hydrophobic interaction of alkane chains from thiol and destabilize the structure, resulting in a higher free Cu^+^ supply. In alkylmonophosphonic acids (MPA), the phosphonate group is occupied with Cu^+^; thus, it does not participate in hydrogen-bonding. Instead, their hydrocarbon chain, when longer than eight carbons, enhances the hydrophobic interaction between hydrocarbon chains of the lamellar framework to increase stability. The high stability of Cu–thiolate ensures a longer induction time of Cu^+^. Thus, an increased deposition of Bi^0^ is expected to transform the liquid Bi-seed into a faceted solid-catalyst. The liquid-seeds enable higher solubility of foreign cations with a tendency to sustain multinucleation steps, thus forming multipods. In contrast, a solid-seed allows limited solubility for foreign cations. Hence it can only afford the formation of a single pod. Therefore, a higher concentration of diphosphonic acid (DPA, > 0.2 mmol) (Figures 3e and S11a) and long (carbon number ≥8) MPA (0.1 mmol) result in single Cu_2-*x*_S pod formation (Figures S6 and S11b). In contrast, a low DPA (0 to 0.1 mmol) concentration reduces the stability of the Cu–thiolate complex, resulting in the formation of Cu_2-*x*_S multipods on Bi (number of pods ∼8 to 11). Additionally, increasing the chain-length of diphosphonic acid reduces the unfavorable interaction between x-type phosphonate groups and the alkane-chain of thiol, forming a mixed population of pods (Figure S11b).

**Figure 3:**
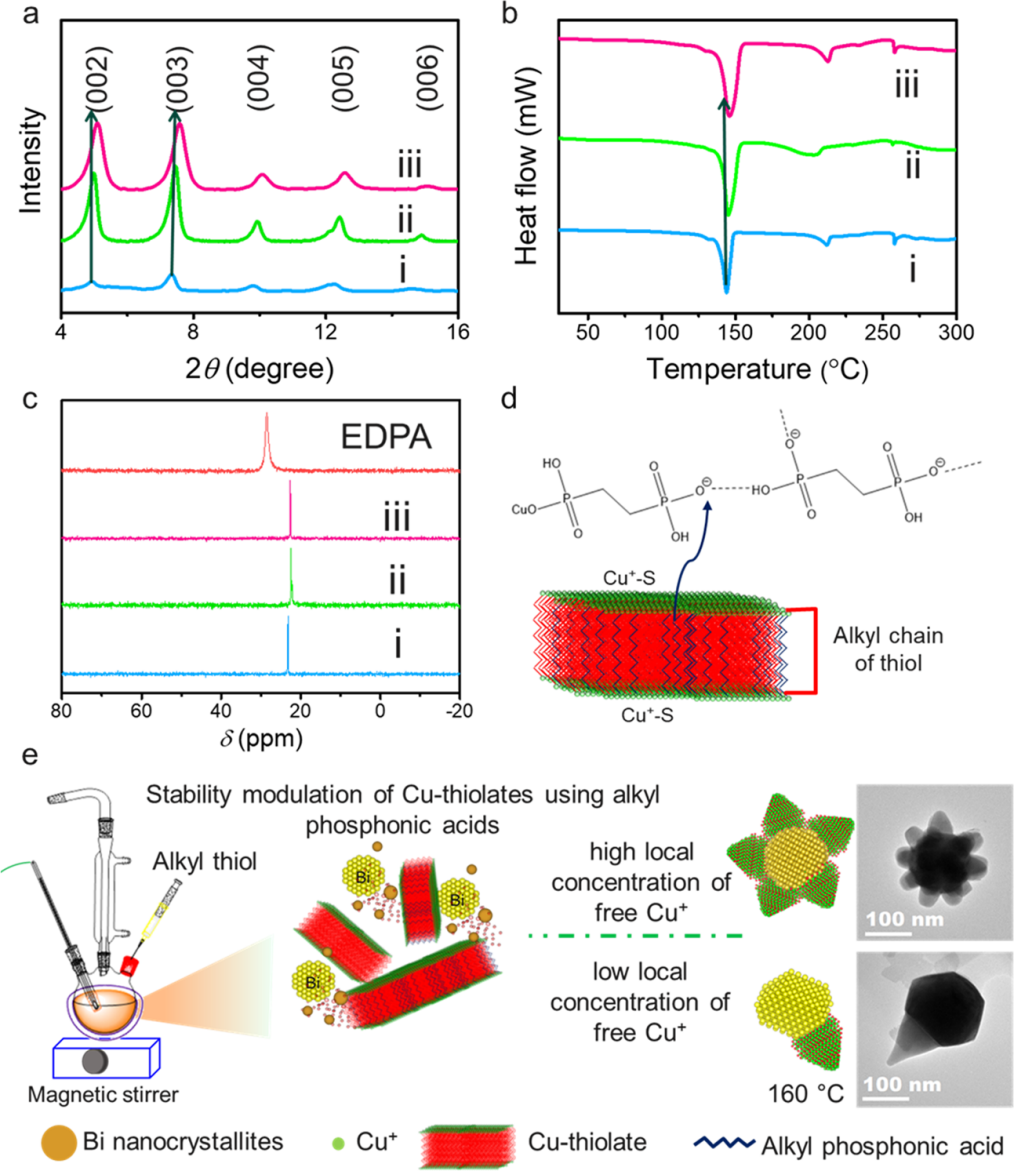
(a) Powder X-ray diffraction (XRD) pattern and (b) thermal analysis using differential scanning calorimetry (DSC) of a Cu–thiolate lamellar complex obtained from 140 °C for EDPA concentrations of (i) 0.1 mmol, (ii) 0.25 mmol, and (iii) 0.5 mmol. (c) ^31^P NMR of the EDPA and the aliquots withdrawn at 140 °C for the reactions with (i) 0.5 mmol, (ii) 0.25 mmol, and (iii) 0.1 mmol of EDPA accompanied by a (d) proposed structure of Cu-EDPA complex present in the Cu–thiolate lamellae. (e) Illustration of the Bi–Cu_2-*x*_S multipods and single pod evolution in solution–liquid–solid growth from insitu generated Bi NCs. In the presence of alkylphosphonic acids, Cu–thiolate controls the Cu^+^ availability during heteronucleation.

The heterostructure NCs were explored as an anode material for K-storage within half cells with K metal as a counter electrode and 4 M potassium bis(fluorosulfonyl) imide in 1,2-dimethoxyethane as the electrolyte. The cyclic voltammograms (CV) obtained at 0.1 mV·s^–1^ between 0.01 and 1.5 V vs K/K^+^ (Figure 4a,b) and between 0.01 and 2.5 V vs K/K^+^ (Figure S7a,b) delineate the multi alloying and dealloying mechanisms of Bi. For the single pod, broad peaks around ∼0.9 in reduction process confirms the potassiation process of Bi (Figure 4a).^44^ For the multipods, the appearance of peaks at ∼0.9, 0.4, and 0.2 V corroborates the potassiation process for multipods, which proceeds via a K_3_Bi_2_ intermediate following Bi ↔ KBi_2_ ↔ K_3_Bi_2_ ↔ K_3_Bi (Figure 4b).^45^ The peak at ∼1.5 V for MP during the first potassiation could be attributed to the irreversible conversion reaction Cu_2-*x*_S.^46^ The single pod electrode display peaks at ∼0.6, 0.7, and 1.2 V during the oxidation process, corresponding to the dealloying process to be K_3_Bi ↔ K_3_Bi_2_ ↔ KBi_2_ ↔ Bi. Overall, CV of multipods suggests enhanced reversibility and reaction kinetics compared to the single pods possibly due to the compactness of the multipod matrix. Figure S8 shows the differential capacity plots (DCP) extracted from galvanostatic voltage profiles for single pod and multipods at different voltage ranges. The DCP of single pod and multipods shows similar trends to CV, and the reduction peak at ∼1.0 V in the DCPs can be attributed to the formation of SEI due to electrolyte decomposition.^47,48^ Irreversibility of the conversion of Cu_2-*x*_S forming Cu is further corroborated by absence of any discharging peak between 1.5 and 2.2 V. The reduction peaks ∼1.2 V (Figure S8a) and 1.5 V (Figure S8b) suggest an irreversible conversion reaction of the metal oxide layer formed on the NC surface and potassiation of Cu_2-*x*_S respectively.^49^ Even for the electrodes cycled between 0.01 and 2.5 V vs K/K^+^, a reversible conversion process of Cu back to Cu_2-*x*_S is observed only in the first few cycles at 2.1 V. Galvanostatic charge–discharge was performed between 0.01 and 1.5 V vs K/K^+^ (Figure 4c,d) and between 0.01 and 2.5 V vs K/K^+^ (Figure S7c,d) at 50 mA·g^–1^ for the initial three cycles, followed by 100 mA·g^–1^ for the rest of the cycles. In the charge–discharge profiles of multipods (Figure 4d), the plateaus at ∼1.2 V and ∼0.5–1 V for the discharging process confirm our observations of K–Bi alloying and dealloying. However, the plateaus observed for single-pod-based electrode are not as pronounced, indicating an inferior kinetics (Figure 4c). For the multipods, after initial 5 cycles, a stable specific capacity of ∼231 mAh·g^–1^ is achieved and displayed a specific capacity of ∼170 mAh·g^–1^ after 200 cycles (Figure 4e). In contrast, the single pod experienced a significant capacity fade to exhibit a capacity of ∼111 mAh·g^–1^ after 200 cycles. The multipods show superior performance with average Coulombic efficiency of 98.1% (0.01–1.5 V) (Figure 4e), even at higher voltage window (Figure S9). Furthermore, the multipods-based anode displays a better rate capability performance at varying current densities ranging from 50 to 800 mA·g^–1^ (Figure 4f).

**Figure 4:**
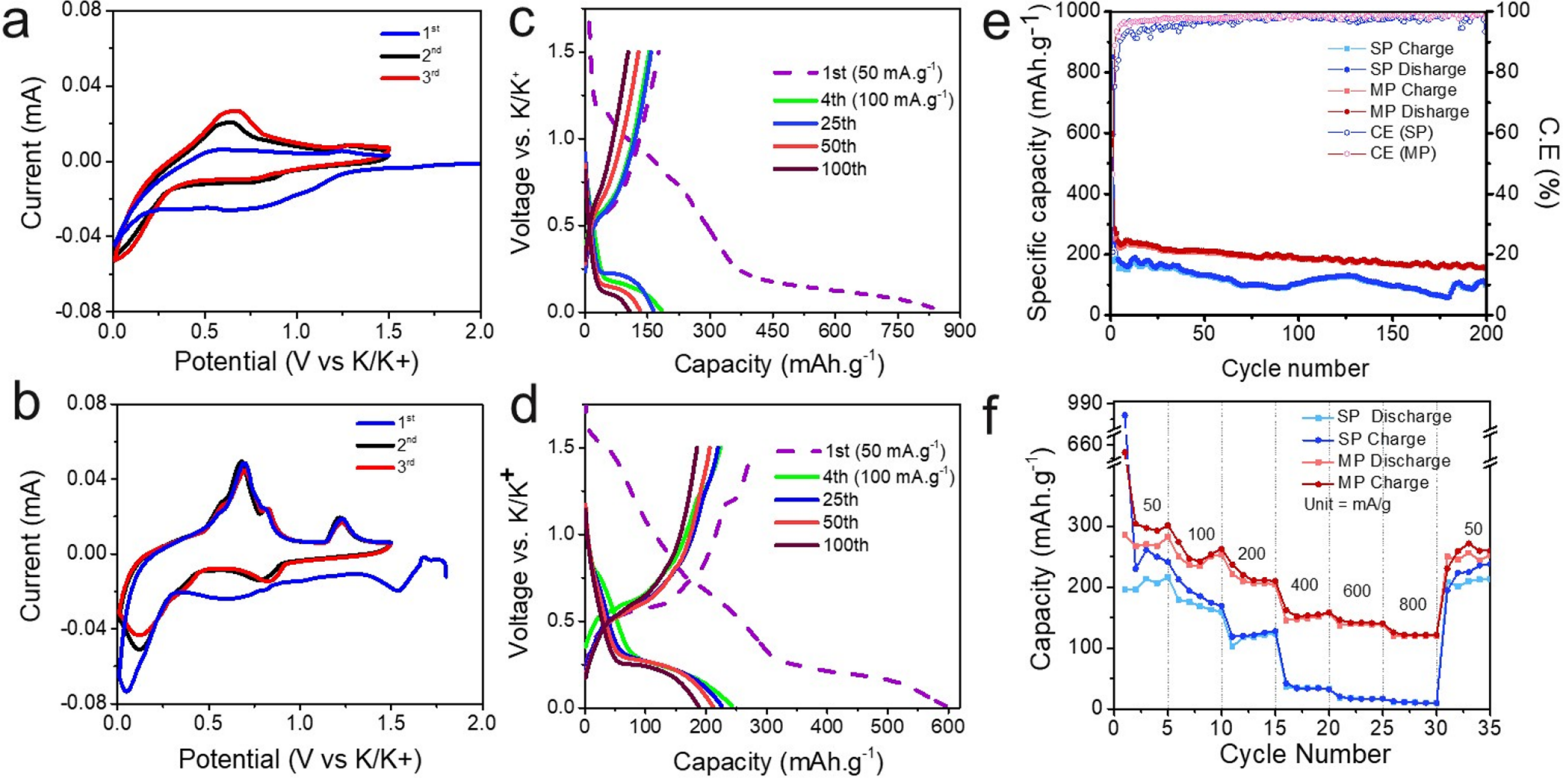
Electrochemical performance of the Bi–Cu_2-*x*_S single pod (SP) and multipod (MP)-based anodes. Cyclic voltammograms acquired at 0.1 mV·s^–1^ between 0.01 to 1.5 V vs K/K^+^ for the (a) SP- and (b) MP-based electrodes, galvanostatic charge–discharge capacity profiles of (c) the SP-based electrode and (d) the MP-based electrode, (e) comparison of cycling performances of SP- and MP-based anodes, and (f) comparison of rate capability performances of SP- and MP-based anodes obtained at different current densities.

To understand the cycling stability difference of the multipod- and single-pod-based electrodes, post 5 and 50 cycle morphologies were analyzed using *ex-situ* scanning electron microscopy (SEM) and TEM (Figure 5a–h and Figure S10). Due to the large volume expansion of Bi (∼400%), the single pods disintegrate as a Cu_2-*x*_S single stem is not capable of containing the seed deformity, which is discernible from SEM images of single pod electrodes after 50 cycle (Figure 5b). The selected area electron diffraction (SAED) pattern of the single-pod-based electrode exhibits complete dealloying of Bi, indicated by the presence of Bi(104) and Bi(205) lattice planes (Figure 5c). The absence of any Cu_2-*x*_S or Cu reflection in the SAED pattern and the presence of Cu and S signals in the ADF-EDS mapping (Figure 5d) suggest the formation of an amorphous Cu–S network upon the potassiation of Cu_2-*x*_S in the first cycle. The multipods retain a spherical envelop like morphology after 50 cycles (Figure 5e,f). A similar observation of the presence of crystalline Bi and absence of Cu-based species from the SAED pattern of the multipods (Figure 5g) and the presence of Cu, Bi, and S signals in ADF-EDS maps of multipods (Figure 5h) demonstrate the Cu–S amorphous network formation with fragmented Bi NCs. Our observation suggests that the buffer network formed from the multiple Cu_2-*x*_S stems in multipods is advantageous to encase the fragmented Bi.

**Figure 5:**
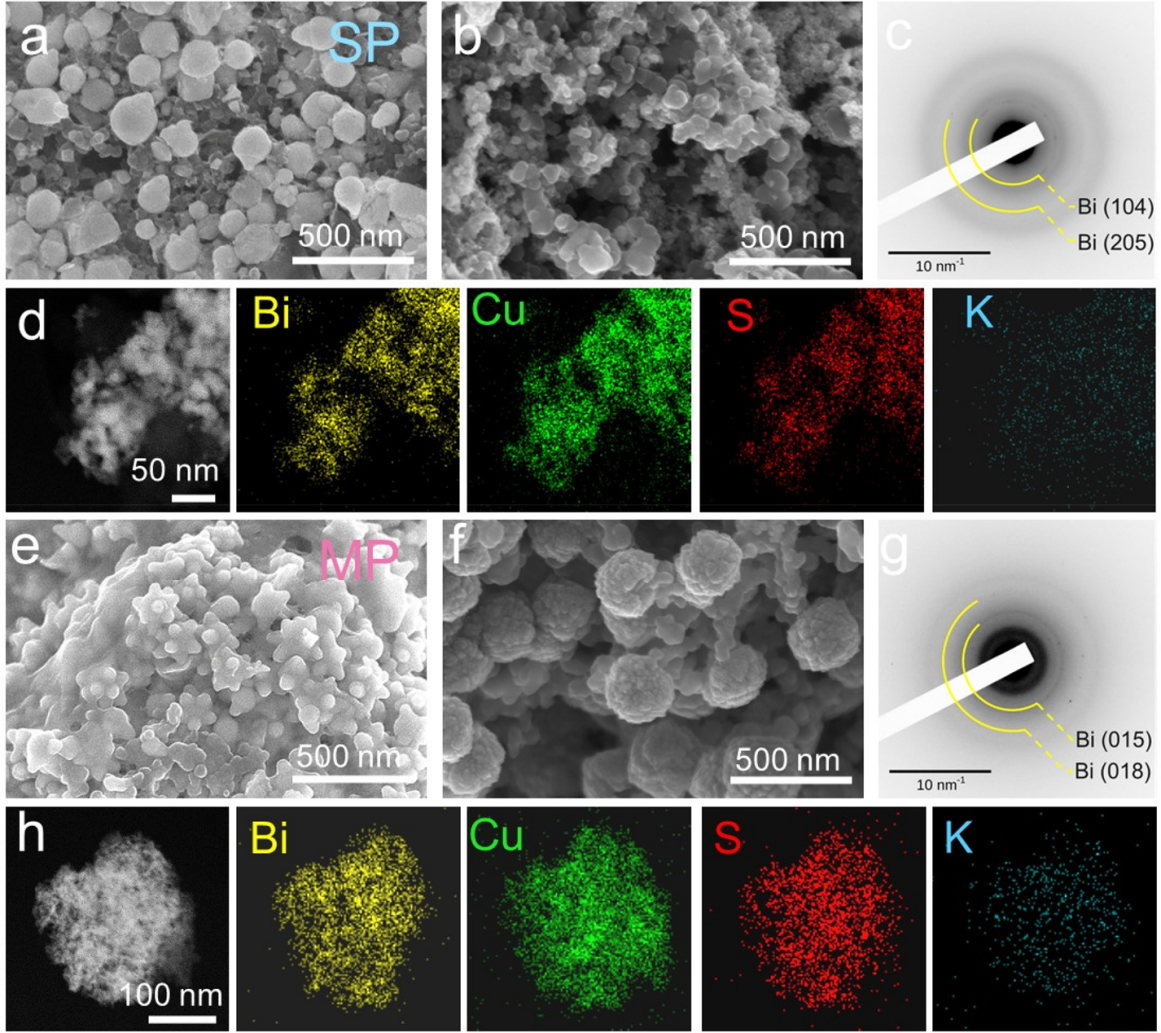
SEM images of (a) pristine single pod (SP) based electrode, (b) electrode after 50 cycles in discharged state; (c) SAED pattern, (d) ADF-EDS maps showing Bi, Cu, S, and K elemental signal from the electrode after 50 cycles in discharged state (0.01 to 1.5 V vs K/K^+^). SEM images of (e) pristine multipods (MPs) based electrode, (f) electrode after 50 cycles in discharged state; (g) SAED pattern, (h) ADF-EDS maps showing Bi, Cu, S, and K elemental signal from the electrode after 50 cycles in discharged state (0.01 to 1.5 V vs K/K^+^).

In summary, we demonstrate a direct colloidal synthesis starting from *in-situ* Bi-seed formation to synthesize Bi(Cu_2-*x*_S)_*n*_ heterostructures with a tunable number of Cu_2-*x*_S stems. We unravel that the local Cu^+^ source Cu–thiolate can be stabilized by adding alkylphosphonic acids to affect the heteronucleation outcome. By increasing the amount of ethylenediphosphonic acid, Cu–thiolate stability can be increased to slow down the Cu^+^ induction rate during heteronucleation forming Bi–Cu_2-*x*_S single pods. Similarly, long-chain alkylmonophosphonic (*n* ≥ 8) acid in low concentration of 0.1 mmol is optimum to stabilize the Cu–thiolate to form single pods. However, a low ethylene diphosphonic acid concentration (0.1 mmol) is crucial to destabilize the Cu–thiolate to form multipods. Short-chains and higher denticity of phosphonic acids are essential for Cu–thiolate instability. Thus, regulation of Cu–thiolate stability with a systematic variation of phosphonic acid ligands is the key to tune the number of Cu_2-*x*_S pods formed. When fabricated as KIB half-cell anodes, the multipods displayed superior rate capability and higher cycling stability compared to the single pods. The *ex-situ* investigation of these heterostructures-based anodes reveals the amorphous network created by conversion of multiple Cu_2-*x*_S stems encasing the fragmented Bi core. Our findings also highlight the importance of foreign cation source regulation to control nucleation in seeded-growth systems.

## Supporting Information Available

Experimental details, additional data of TEM, NMR analysis of the aliquot samples, and additional data of potassium ion storage performance (PDF)
