# Supplementary material for: Multipod Bi(Cu2-xS)n Nanocrystals formed by Dynamic Cation–Ligand Complexation and Their Use as Anodes for Potassium-Ion Batteries
Source: Nano Lett. 2022 Dec 6;22(24):10120–7. doi: 10.1021/acs.nanolett.2c03933 (PMC9801429; doi:10.1021/acs.nanolett.2c03933)
Supplement: Supplementary file 1 — nl2c03933_si_001.pdf [file nl2c03933_si_001.pdf]

Multipod Bi-$(Cu_{2-x}S)_n$ Nanocrystals formed by Dynamic Cation-Ligand Complexation and their use as Anodes for Potassium-Ion Batteries

Nilotpal Kapuria, Sumair Imtiaz, Abinaya Sankaran, Hugh Geaney, Tadhg Kennedy, Shalini Singh, Kevin M Ryan**

Email: Kevin.M.Ryan@ul.ie, Shalini.Singh@ul.ie

Department of Chemical Sciences and Bernal Institute, University of Limerick, V94T9PX Limerick, Ireland

Table of content:

1.1. Chemicals 2

1.2. Bi-$Cu_{2-x}S$ nanocrystal (NC) synthesis. 3-4

1.3. NC purification procedure 4

1.4. Aliquot study 4

1.5. Electron Microscopy 5

1.6. X-ray diffraction (XRD) analysis. **5**

1.7. Electrochemical Measurements **5**

1.8. TEM analysis of aliquot collected at 140 °C **6**

1.9. ^{1}H NMR analysis of aliquots **7-8**

1.10. Evolution of Bi-Cu_{2-x}S heterostructures **8**

1.11. TEM image of heterostructures synthesized

using phosphonic acids of different chain length **9**

1.12. Electrochemical analysis of Bi-Cu_{2-x}S-based anodes **10-12**

1.13. Post characterization of Bi-Cu_{2-x}S-based anodes **12**

1.14. Variation of Cu2-xS stems in Bi-Cu_{2-x}S **13**

1.15. Cu-thiolate **13**

1.1. Chemicals. Copper acetylacetonate [$Cu(acac)_2$, 97%, Lot# STBD3281V], Bismuth Chloride ($BiCl_3$, ≥98%, Lot# MKBV5130V), tert-dodecyl mercaptan (*t*-DDT, 98.5%, Lot# STBH4978), 1-dodecanethiol (1-DDT, ≥ 98%, Lot# STBF43147V), Oleyl amine (OLA, 70%, Lot# STBJ0354), 1-Octadecence (ODE, 90%, Lot# NKBL4740V), hexylphosphonic acid (97%, HPAA15180212), were purchased from Sigma Aldrich. Toluene (Tol), Methanol and Ethylacetate were purchased from Lennox, Ireland. 1,2 ethtylenediphoaphonic acid (EDPA, 97%, Lot# 10212828), was purchased from Alfa Aesar. The 1,6 hexylenediphoaphonic acid (HDPA, >98%, OMQTJ) was purchased from TCI. n-Octyl phosphonic acid (OPA, Lot# 051-99) was purchased from PCI. n-Tetradecyl phosphonic acid (TDPA, 97%, Lot# AK1803131) was purchased from Plasmachem. The chemicals were used as received without any further purification.

1.2. Bi-$Cu_{2-x}S$ nanocrystal (NC) synthesis. In a typical synthesis, 130.9 mg (0.5 mmol) $Cu(acac)_2$, 157.7 mg (0.5 mmol) $BiCl_3$, 0.1-0.5 mmol of alkyl phosphonic acid/ diphosphonic, were mixed with 2 ml OLA and 8 ml ODE solvent mixture in a 3-neck round bottom flask (RBF) and reaction mixture was evacuated at 105 °C for 25 min (5 min ramp to 105 °C and 25 min soak). The vacuum pressure was kept below 200 mTorr during evacuation. Afterwards, the reaction mixture was heated to 160 °C under an argon atmosphere (3 min ramp to 160 °C). 1ml of thiol mixture (0.875 ml 1-DDT and 0.125 ml *t*-DDT) was injected when the temperature reached 135 °C. After thiol injection the reaction mixture turned to bright orange from blue and finally turned to black above 147 °C (Figure S1). When the temperature of reaction vessel reached 160 °C it was allowed to proceed for another 5 min of growth time. Afterwards the heating mantle was removed to terminate the reaction by natural cooling till 80 °C. Upon reaching 80 °C, 8 ml of Ethylacetate was injected to quench the reaction. For all experiments 0.1 mmol of alkyl phosphonic acid is used unless mentioned otherwise.

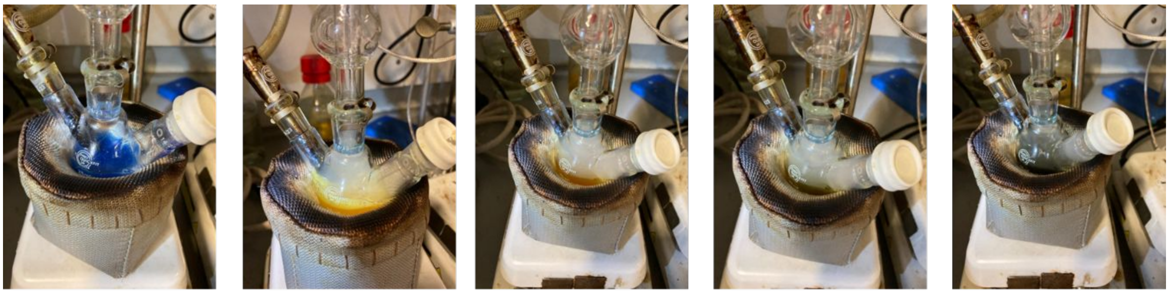

Figure S1. The reaction mixture color at different temperatures.

1.3. NC purification procedure. The heterostructures synthesized and quenched with 8 ml of Ethylacetate were poured into a 50 ml centrifuge tube and vortexed well. After that, the NC solution was centrifuged at 5000 rpm for 5 min. The pellet was collected and dispersed in 10 ml of Tol, and 10 ml of Ethylacetate was further added and vortexed to disperse the NCs well. The NC solution was again centrifuged at 5000 rpm for 5 min and the process was repeated another 2 times and dried at 80 °C overnight in vacuum.

1.4. Aliquot study. During NC growth 1 ml solution from the RBF was withdrawn at desired temperature and time after thiol injection. To ensure minimal depletion in precursor concentration a maximum of 2 ml of reaction solution in total was withdrawn from RBF. After withdrawal, the growth was immediately quenched by ejecting into 2 ml of Tol. The NCs in 2ml of Tol were dispersed in 2ml of IPA and centrifuged for 5 min at 5000 rpm. Followed by another two cycles of redispersion in 2 ml Tol and 2ml IPA and centrifugation at 5000 rpm for 3 min. For isolating the Cu-thiolate complex the aliquot collected at 140 ˚C is centrifuged at 8000 rpm. The supernatant is separated and mixed with 5 ml of methanol to isolate the Cu-thiolate as precipitate and dried to obtain an orange color powder and stored in glovebox for further characterization (Figure S12). For NMR characterization

the aliquot collected at 140 °C was thermally quenched and no toluene was added. After cooling it down to room temperature the liquid portion of the aliquot characterized through ^{1}H NMR and ^{31}P NMR (JEOL 400 MHz NMR spectrometer) in $CDCl_3$. The peaks were referenced to the residual chloroform peak at 7.26 ppm for ^{1}H NMR.

1.5. Electron Microscopy. For transmission electron microscopy (TEM) analysis the NCs were dispersed in Tol and drop casted on continuous carbon coated 200 mesh nickel grids. Low resolution and high-resolution TEM (HRTEM) and dark-field scanning transmission electron microscopy (DFSTEM) was conducted by using a 200 kV JEOL JEM-2100F field emission microscope, equipped with a Gatan Ultra scan CCD camera and EDAX Genesis energy dispersive x-ray spectroscopy (EDS) detector. For analyzing the HR-TEM data, interplanar distances and particle orientation were determined from the selected area FFT analysis using GMS3 software.

1.6. X-ray diffraction (XRD) analysis. XRD of drop-cast films of the NCs on the flat surface of p-type boron doped silicon zero background was conducted using a PANalytical Empyrean instrument equipped with a Cu Kα radiation source (λ =1.5418 Å) and a 1-D X'celerator strip detector with the diffractometer operating at 40 kV and 40 mA.

1.7. Electrochemical Measurements. To prepare the electrodes, 60 wt.% active material (SP or MP), 30 wt% conductive carbon, and 10 wt% CMC were uniformly mixed by mortar and pestle in deionized water. The as-prepared slurry was coated on Cu foil using doctor-blade and then dried at room temperature for 2 days, followed by drying at 70 ^{o}C for about 12 hrs in oven. The active material loading was in the range of 0.45 – 0.65 $mg.cm^{-2}$. The electrochemical performance of the as synthesized MP and SP anodes were evaluated by assembling CR2032 coin cells in an Ar-filled glove box (< 0.1 ppm O_2 /H_2O). The cells consisted of the MP or SP anodes as the working electrode, potassium metal (99.95 %) as the counter/reference electrode, and glass fiber (GF/D, Whatman®) as a separator. 100 μl

of 4 M KFSI in DME is used as an electrolyte. The current densities were calculated based on the mass of total active material (Bi-$Cu_{2-x}S$). The cell were tested in the voltage window of 0.01 – 2.5 and 0.01 – 1.5 V. Galvanostatic measurements were carried out using a Biologic MPG-2 and the cyclic voltammetry (CV) tests were performed at a scan rate of 0.1 $mV.s^{-1}$.

1.8. TEM analysis of aliquot collected at 140 °C

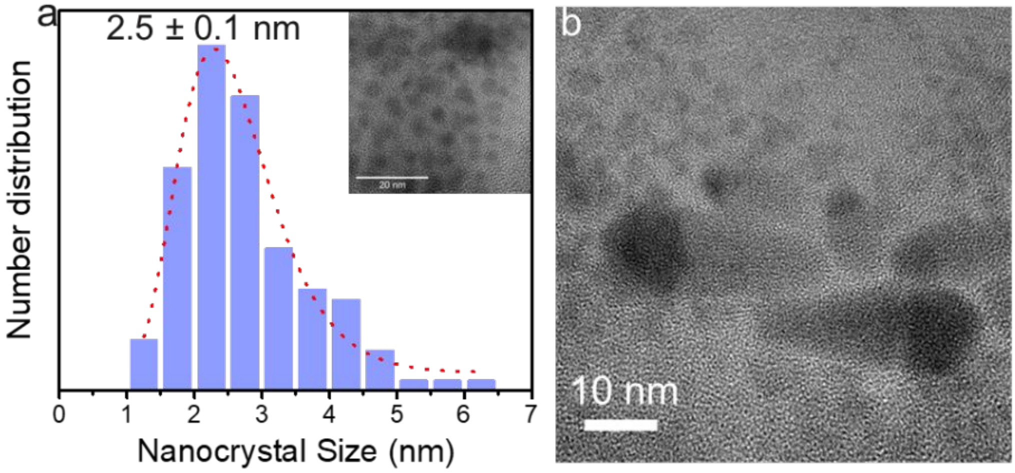

Figure S2. (a) Size distribution of the Bi NCs present in the aliquot 140 ˚C, (b) Co-existing heterostructures and Bi NCs in the aliquot 140 ˚C.

1.9. ^{1}H NMR analysis of aliquots

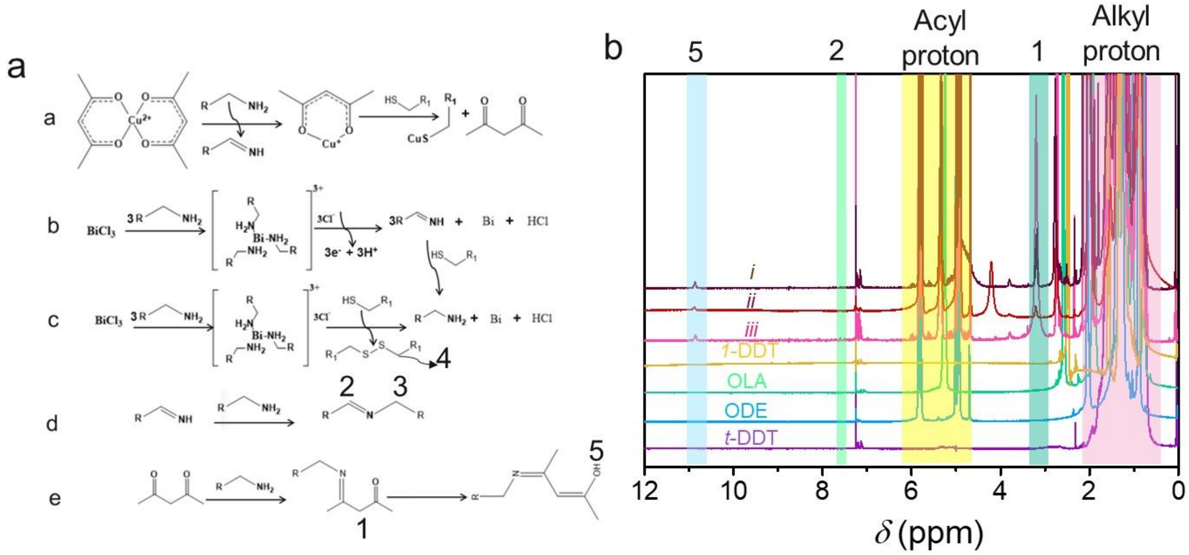

Figure S3. (a) Plausible reaction mechanisms before heteronucleation, (b) ^{1}H NMR of aliquots withdrawn at 140 °C from reactions performed using (i) 0.5, (ii) 0.25, (iii) 0.1 mmol of EDPA, and the solvent used in the reaction in $CDCl_3$, the reference peak of chloroform is at 7.26 ppm. The acyl (~5.4 ppm) and alkyl (~0.6 to 2.1 ppm) protons of the Schiff bases overlap with the acyl and protons of OLA, and ODE making it difficult to be distinguished.

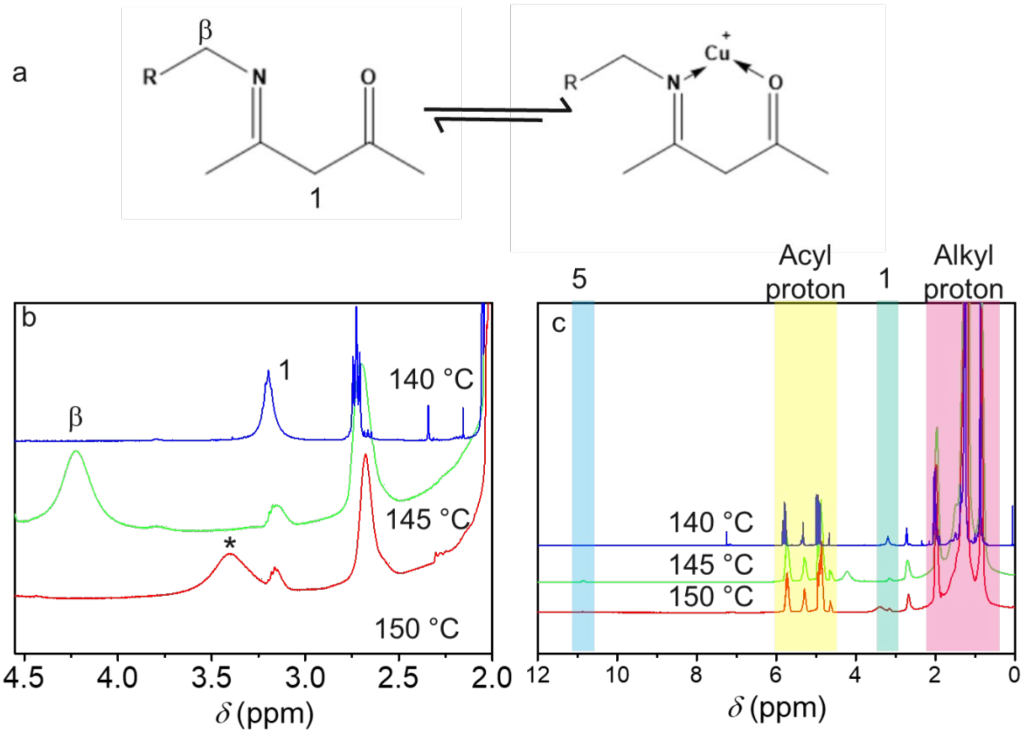

Figure S4. (a) Co-ordination chemistry between ketimine and Cu^{+}. ^{1}H NMR of aliquots withdrawn at 140, 145 and 150 ˚C from reactions performed using 0.1 mmol of EDPA in $CDCl_3$, the reference peak of chloroform is at 7.26 ppm showing peaks from (b) 2-4.5 ppm and (c) 0-12 ppm. The free Cu^{+} generated from melting of Cu-thiolate co-ordinates with ketimine showing a shift of the β-proton peak to ~4.2 ppm with presence of free ketimine showing α-proton peak at ~3.2 ppm as seen for all the aliquots. After the formation of more of the heterostructures the free Cu^{+} concentration depletes which can be suggested by the disappearance of the β proton peak ~4.2 ppm from the 150 ˚C aliquot. Furthermore, a broad peak around ~3.4 ppm appears for the 150 ˚C aliquot. This peak can be described as α-proton peak of either nanocrystal surface bound oleylamine or nanocrystal surface bound ketimine.

1.10. Evolution of Bi-Cu_{2-x}S heterostructures

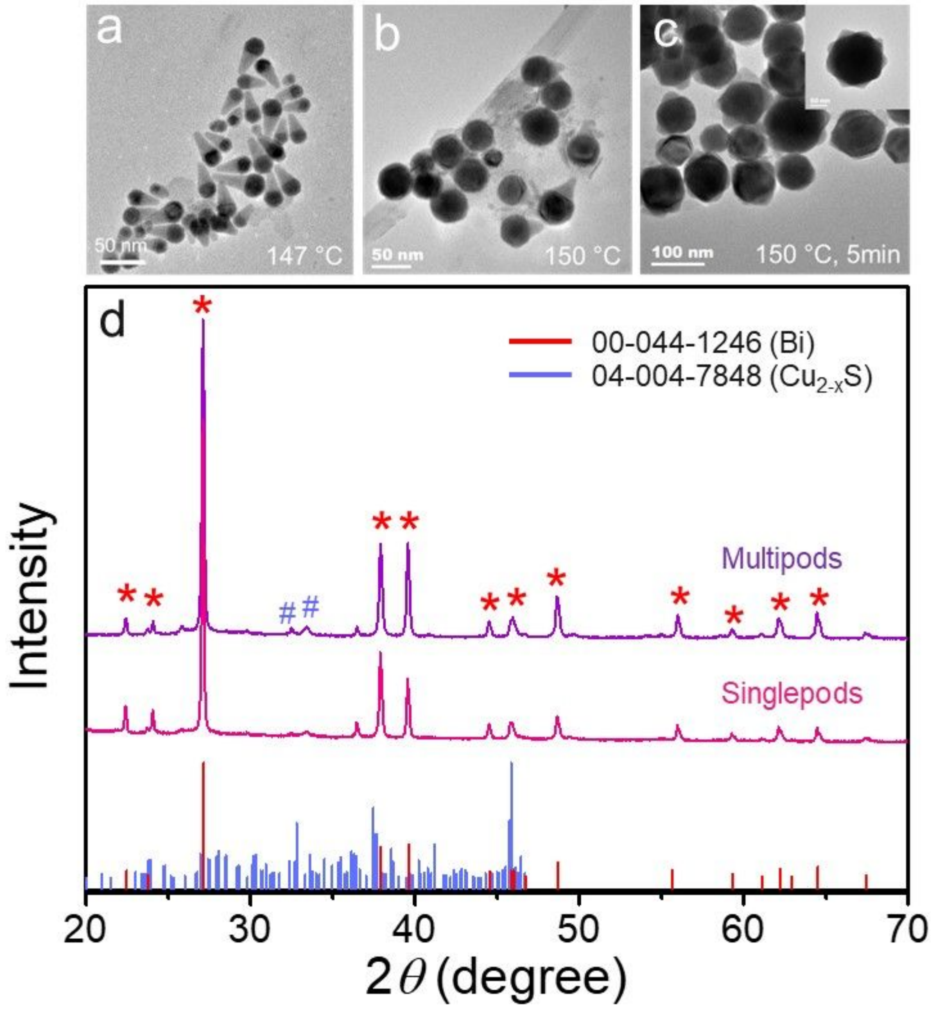

Figure S5. (a) TEM of Bi-$Cu_{2-x}S$ heterostructures obtained at (a) 147 °C, after (a) 30 sec and (b) 5 min at 150 °C; (d) XRD patterns of multipods and singlepods with the corresponding reflection of bulk Bi (PDF number 00-044-1246) and bulk monoclinic $Cu_{2-x}S$ (PDF number 04-004-7848). However, the peak intensity ratio of Bi to $Cu_{2-x}S$ of 6: 1, and the overlapping of main peaks make it hard to get any discernible signals from the chalcocite phase in the powder XRD pattern.

1.11. TEM image of heterostructures synthesized using phosphonic acids of different chain length

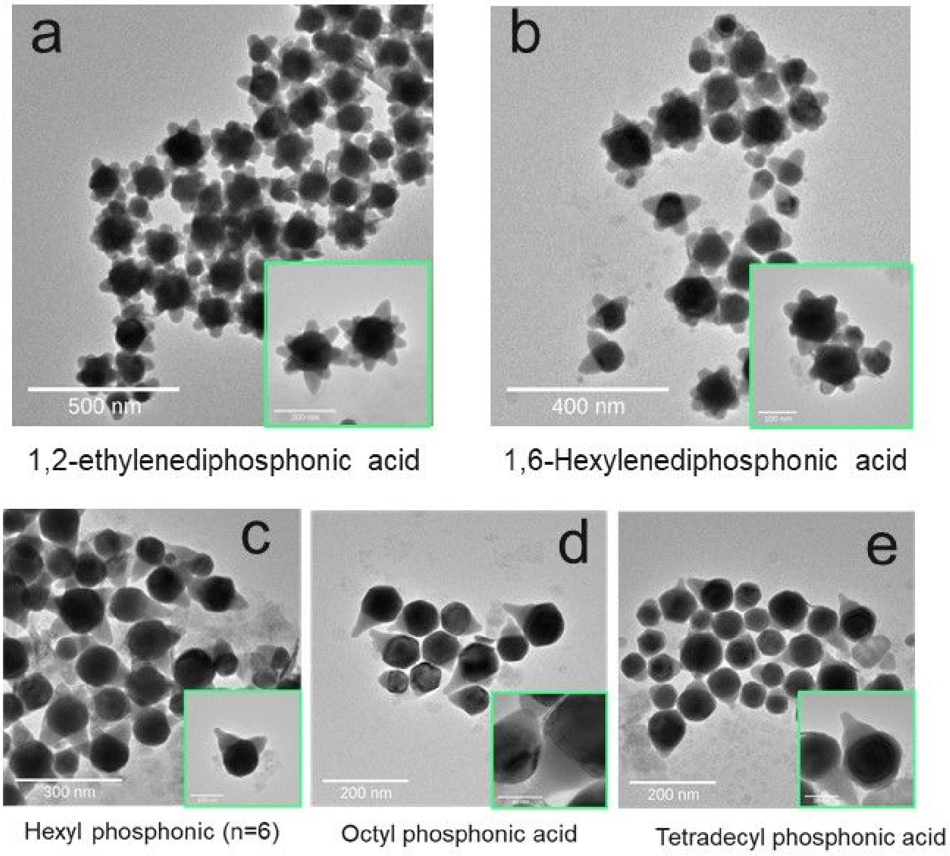

Figure S6. Bi-$Cu_{2-x}S$ heterostructures obtained using (a)1,2-ethylenediphosphonic acid, (b)1,6- hexylenediphosphonic acid, (c) hexyl phosphonic acid, (d) octyl phosphonic acid, (e) tetra decylphosphonic acid.

1.12. Electrochemical analysis of Bi-$Cu_{2-x}S$-based anodes

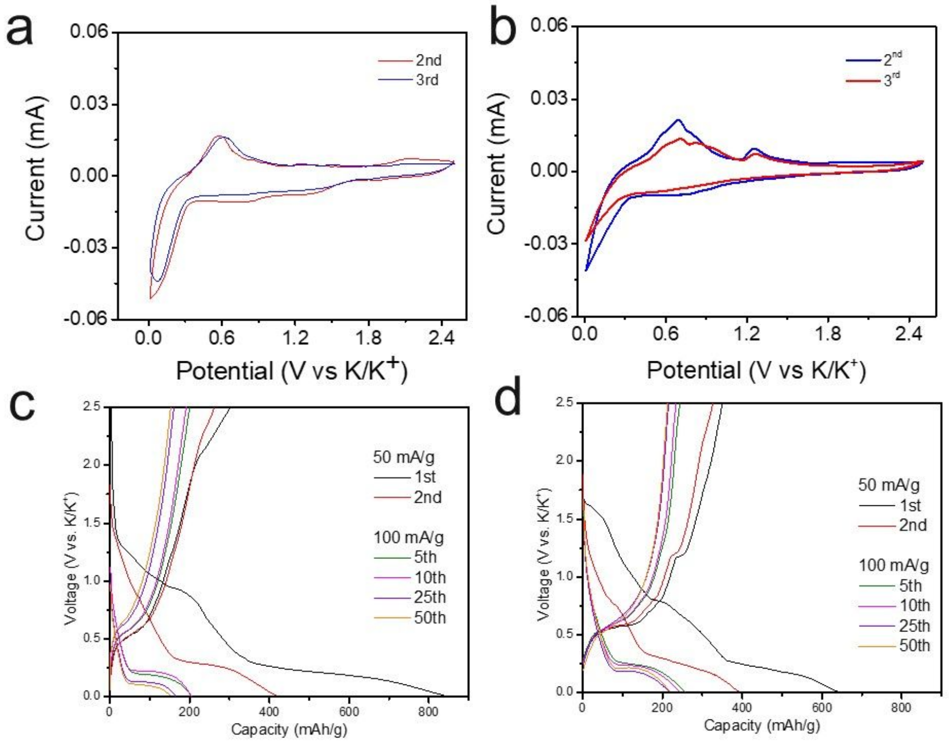

Figure S7. Cyclic voltammograms acquired at 0.1 mV.s^{-1} between 0.01 to 2.5 V vs K/K^{+} for the (a) singlepods (SPs), (b) multipods (MPs) based electrodes and galvanostatic charge discharge profiles of (c) SPs, (d) MPs between 0.01 to 2.5 V vs K/K^{+}.

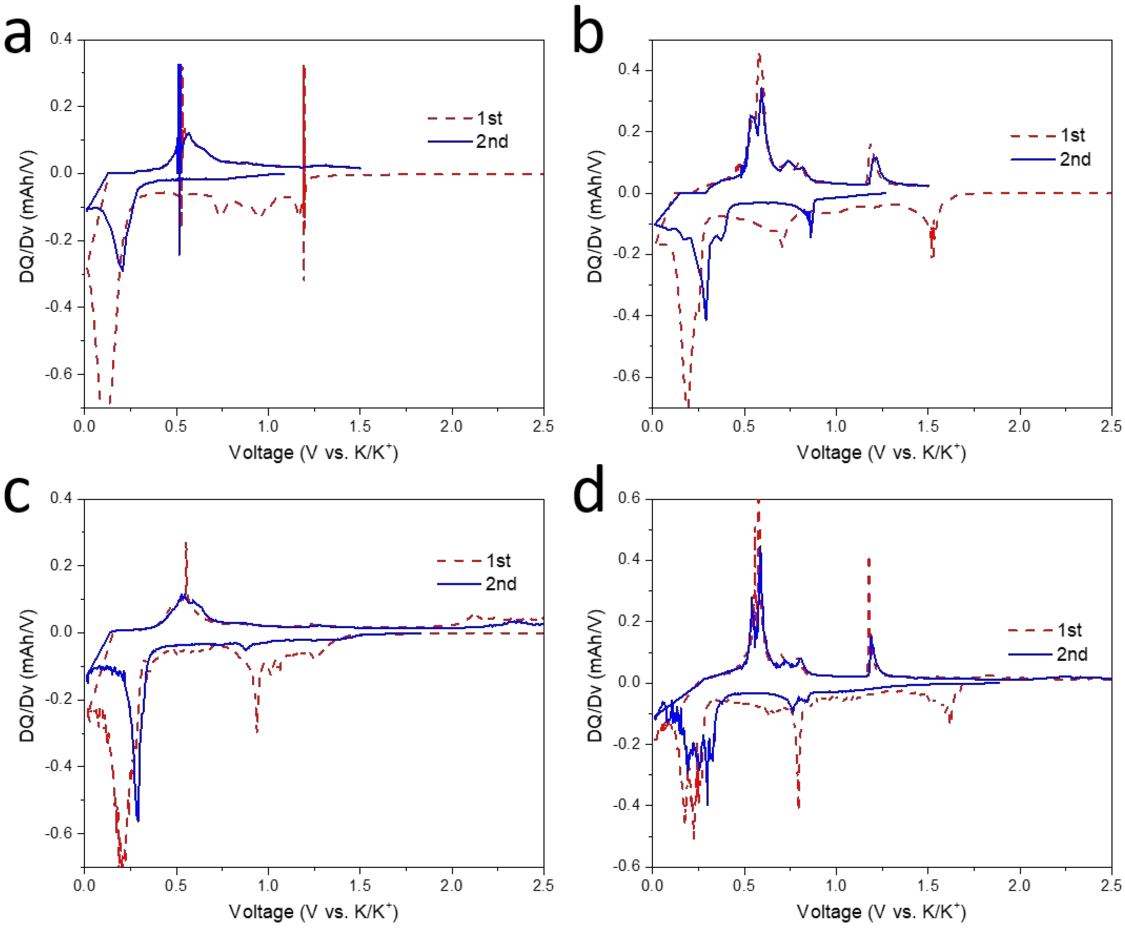

Figure S8. Differential capacity plot for the 1st and 2nd cycle obtained for (a) singlepod (SP), (b) multipods (MP) based electrode between 0.01 to 1.5 V vs K/K^+ and for (c) SP, (d) MP based electrode between 0.01 to 2.5 V vs K/K^+.

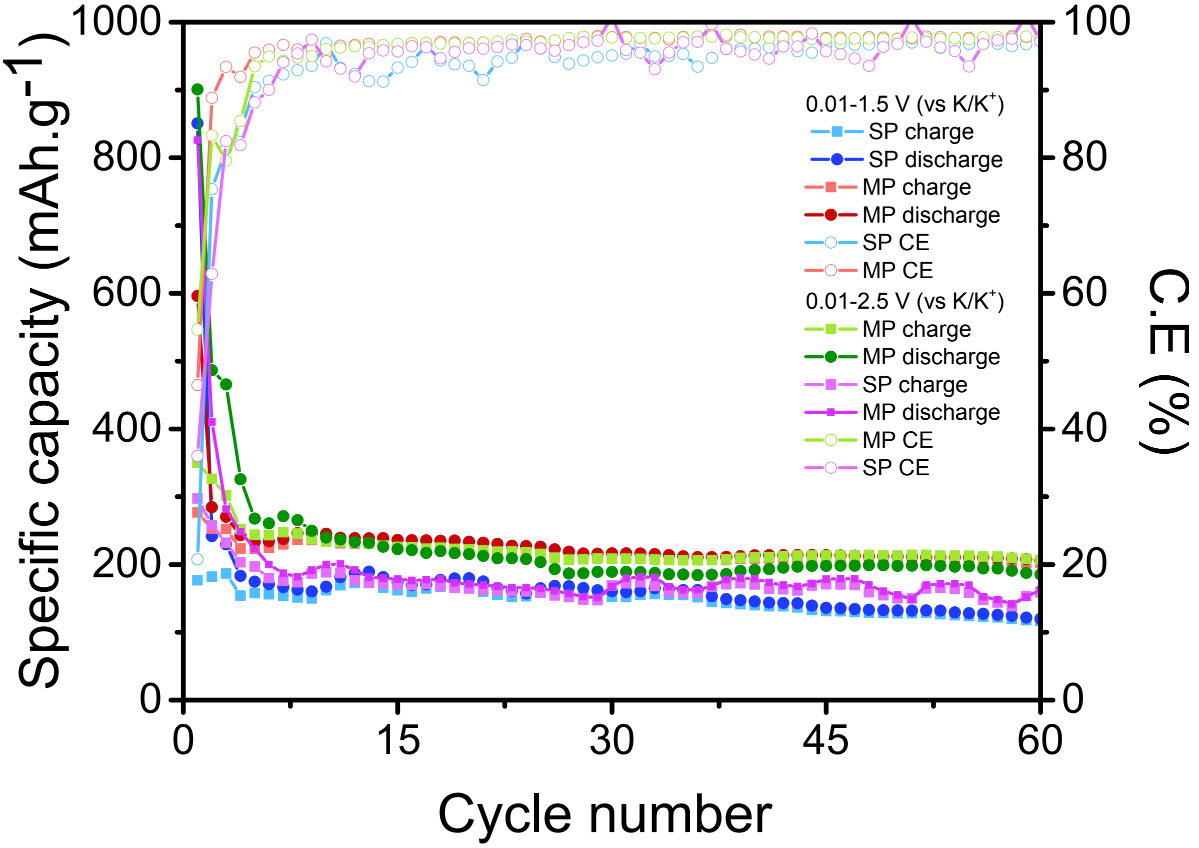

Figure S9. Comparison of cycling performances of singlepod (SP) and multipod (MP)-based anodes at different voltage ranges.

1.13. Post characterization of Bi-$Cu_{2-x}S$-based anodes

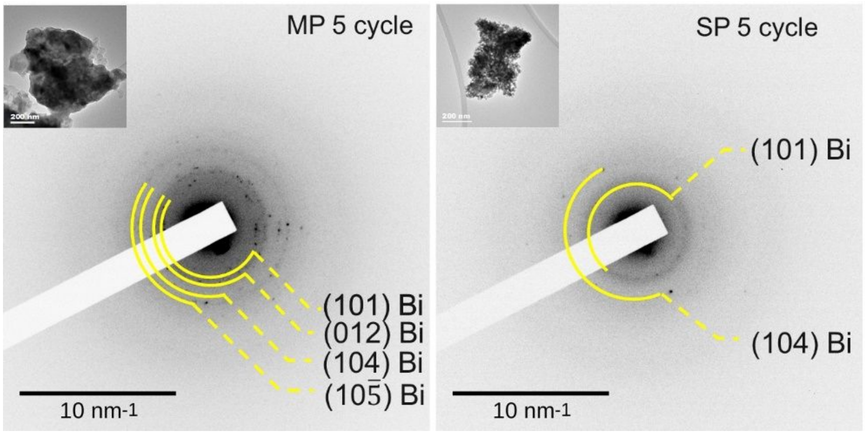

Figure S10. TEM image (inset) and SAED pattern of Multipod (MP) based electrode (left), and single pod (SP) based electrode (right) after 50 cycles in discharged state (0.01 to 1.5 V vs K/K^+).

1.14. Variation of $Cu_{2-x}S$ stems in Bi-$Cu_{2-x}S$

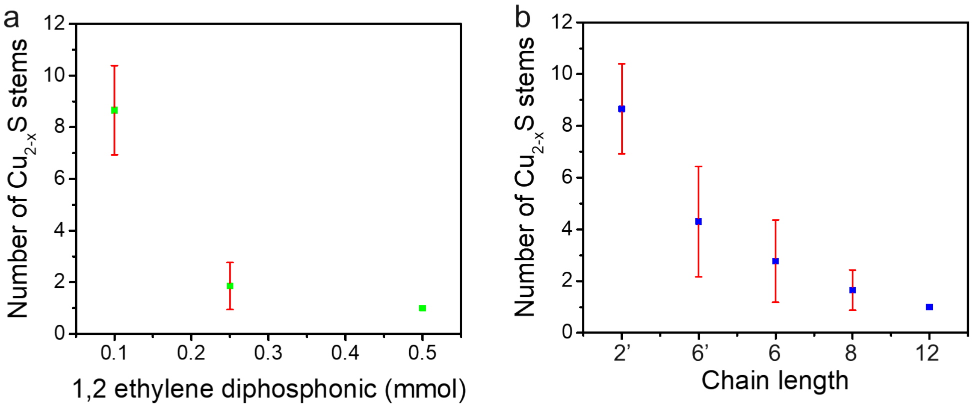

Figure S11. Variation of number of $Cu_{2-x}S$ stems based on (a) 1,2 ethylenediphosphonic acid concentration, (b) phosphonic acid chain length (2' =1,2 ethylene diphosphonic acid, 6'= 1,6 hexylenediphosphonic, 6= hexyl phosphonic acid, 8=octyl phosphonic acid, 12= tetradecyl phosphonic acid).

1.15. Cu-thiolate

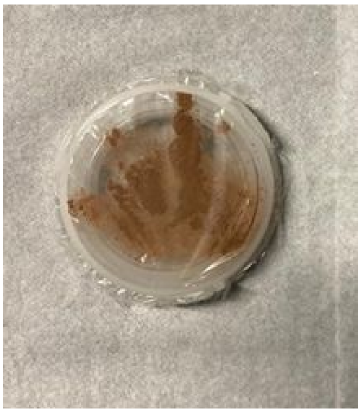

Figure S12. Isolated Cu-thiolate.
